# Abnormal methylation of seven genes and their associations with clinical characteristics in early stage non-small cell lung cancer

**DOI:** 10.3892/ol.2013.1161

**Published:** 2013-01-29

**Authors:** YANGXING ZHAO, HUAFU ZHOU, KELONG MA, JINFENG SUN, XU FENG, JUNFENG GENG, JUN GU, WEI WANG, HONGYU ZHANG, YINGHUA HE, SHICHENG GUO, XIAOYU ZHOU, JIAN YU, QIANG LIN

**Affiliations:** 1 State Key Laboratory of Oncogenes and Related Genes, Shanghai Cancer Institute, Renji Hospital, Shanghai Jiao Tong University School of Medicine, Shanghai 200032;; 2 Department of Cardiac Surgery, The First Affiliated Hospital of Guangxi Medical University, Nanning 530022;; 3 Department of General Thoracic Surgery, Shanghai Chest Hospital, Shanghai Jiao Tong University, Shanghai 200030;; 4 Ministry of Education Key Laboratory of Contemporary Anthropology School of Life Sciences, Fudan University, Shanghai 200443;; 5 Key Laboratory of Contraceptive Drugs and Devices of NPFPC, Shanghai Institute of Planned Parenthood Research, Shanghai 200032;; 6 School of Life Science, Anhui Medical University, Hefei 230032;; 7 School of Integrated Traditional Chinese and Western Medicine, Anhui University of Traditional Chinese Medicine, Hefei 230038, P.R. China

**Keywords:** DNA methylation, non-small cell lung cancer, stage I, smoking

## Abstract

To identify novel abnormally methylated genes in early stage non-small cell lung cancer (NSCLC), we analyzed the methylation status of 13 genes (ALX1, BCL2, FOXL2, HPP1, MYF6, OC2, PDGFRA, PHOX2A, PITX2, RARB, SIX6, SMPD3 and SOX1) in cancer tissues from 101 cases of stage I NSCLC patients and lung tissues from 30 cases of non-cancerous lung disease controls, using methylation-specific PCR (MSP). The methylation frequencies (29.70–64.36%) of 7 genes (MYF6, SIX6, SOX1, RARB, BCL2, PHOX2A and FOLX2) in stage I NSCLC were significantly higher compared with those in non-cancerous lung disease controls (P<0.05). The co-methylation of SIX6 and SOX1, or the co-methyaltion of SIX6, RARB and SOX1 was associated with adenosquamous carcinoma (ADC), and the co-methylation of BCL2, RARB and SIX6 was associated with smoking. A panel of 4 genes (MYF6, SIX6, BCL2 and RARB) may offer a sensitivity of 93.07% and a specificity of 83.33% in the diagnosis of stage I NSCLC. Furthermore, we also detected the expression of 8 pathological markers (VEGF, HER-2, P53, P21, EGFR, CHGA, SYN and EMA) in cancer tissues of stage I NSCLC by immunohistochemistry, and found that high expression levels of p53 and CHGA were associated with the methylation of BCL2 (P=0.025) and PHOX2A (P=0.023), respectively. In this study, among the 7 genes which demonstrated hypermethylation in stage I NSCLC compared with non-cancerous lung diseases, 5 genes (MYF6, SIX6, PHOX2A, FOLX2 and SOX1) were found for the first time to be abonormally methylated in NSCLC. Further study of these genes shed light on the carcinogenesis of NSCLC.

## Introduction

In China, the incidence of lung cancer is increasing every year, and the mortality rate of lung cancer has risen to the highest among all types of cancer (1). The main type of lung cancer is non-small cell lung cancer (NSCLC), which accounts for 85% of all lung cancer cases.

Abnormalities in epigenetics are known to play important roles in the development and progression of cancer. Changes in DNA methylation patterns, common events in cancer cells, may result in disturbance of the expression of genes that control cell proliferation, differentiation and apoptosis, and lead to carcinogenesis. Furthermore, abnormalities in DNA methylation are promising diagnostic markers for the early detection of cancer (2).

In this study, cancer tissues from 101 patients with stage I NSCLC and lung tissues from 30 patients with non-cancerous lung diseases, were detected for the methylation status of 13 genes: PITX2 (paired-like homeodomain 2), RARB (retinoic acid receptor, β), OC2 (one cut homeobox 2), MYF6 (myogenic factor 6), PDGFRA (platelet-derived growth factor receptor, α polypeptide), SOX1 [SRY (sex determining region Y)-box 1], ALX1 (ALX homeobox 1), SIX6 (SIX homeobox 6), PHOX2A (paired-like homeobox 2a), FOXL2 (forkhead box L2), SMPD3 (sphingomyelin phosphodiesterase 3), BCL2 (B-cell CLL/lymphoma 2) and HPP1 (hyperpigmentation, progressive, 1). The methyaltion frequencies of 7 genes: MYF6, SIX6, SOX1, RARB, BCL2, PHOX2A and FOLX2 were significantly higher in stage I NSCLC than in non-cancerous lung diseases.

## Materials and methods

### Clinical tissue samples

The clinical tissue samples from 101 stage I NSCLC patients and 30 patients with non-cancerous lung diseases used in this study were obtained from the Shanghai Chest Hospital (Shanghai, China) and the First Affiliated Hospital of Guangxi Medical University (Nanning, China). Informed consent was obtained from the patients and the study was approved by the Medical Institutional Review Boards of the two hospitals. Tumor-node-metastasis (TNM) staging/classification of the patients was performed according to the WHO classification. Table I shows the clinical patient profiles.

### DNA isolation and methylation-specific PCR (MSP)

Genomic DNA of clinical tissues were isolated by a standard phenol/chloroform purification method. The primers for MSP were designed according to: http://www.urogene.org/methprimer/index1.html (Table II). The bisulfate conversion and PCR analysis were conducted as previously described (3).

### Immunohistochemical analysis

Among the 101 cases of stage I NSCLC patients, 92 routinely underwent detection of 8 pathological protein markers by immunohistochemistry before chemotherapy. These pathological markers were: vascular endothelial growth factor (VEGF), human epidermal growth factor receptor 2 (HER-2), p53, p21, epidermal growth factor receptor (EGFR), chromogranin A (CHGA), synaptophysin (SYN) and epithelium membrane antigen (EMA). For each sample, the H&E-stained sections were first reviewed and marked for the selected point. Tumor samples were embedded in paraffin and cut into 3-μm sections. Sections were processed using the Super Sensitive Link-Labeled Detection System (Biogenex, Menarini, Florence, Italy). The first antibodies used in this study were purchased from Sant Cruz Biotechnology (Santa Cruz, CA, USA). The second antibodies were purchased from KangChen Bio-Tech Inc. (Shanghai, China). The enzymatic activity was developed using 3-amino-9-ethylcarbazole (Dako, Milan, Italy) as a chromogenic substrate. The result was scored by conjunction with both staining intensity and the percentage of positive staining cells. Each sample was given an intensity score (0–3) and a percentage of cell positive score (0, <5%; 1, 5–25%; 2, 25–50%; 3, 50–75%; 4, > 75%). An overall immunohistochemistry score was calculated by multiplying the intensity and percentage of cell positive scores. Scores of 1–4 were recorded as +, 6–8 as ++, and 9–12 as +++.

### Statistical analysis

All statistical calculations were performed using the SPSS 13.0 software statistical package (SPSS Inc., Chicago, IL, USA). The incidence of hypermethylation in NSCLC tissues vs. the non-cancerous tissues was calculated using a 2×2 Fisher’s exact test. The associations among the pathological variables and the methylation status of the genes were assessed by means of univariate and multivariate logistic-regression analysis. The area under the receiver operating characteristic (ROC) curve (AUC) is a measure of the ability of a continuous marker to accurately classify tumor tissues and non-tumor tissues. Correlations between the expression of pathological markers and gene methylation were examined using the Chi-square test. P<0.05 was considered to indicate a statistically significant result.

## Results

### Methylation frequencies of the 7 genes differ significantly between stage I NSCLC and non-cancerous controls

Among the 13 genes, the methylation frequencies of 7 genes (MYF6, SIX6, SOX1, RARB, BCL2, PHOX2A and FOLX2) had significant difference between the group of stage I NSCLC and the group of non-cancerous lung diseases (Table III). ROC curves were constructed for each of the 7 genes to classify stage I NSCLC and non-cancerous lung disease. The AUC of the ROC curve for MYF6 was 0.704 (P<0.0001; 95% CI, 0.613–0.795), which was the largest among the 7 genes. The sensitivity and specificity of MYF6 were 64.36 and 93.33%, respectively, in the diagnosis of stage I NSCLC. The AUC of the ROC curves for the other 6 genes (SIX6, SOX1, RARB, BCL2, PHOX2A and FOLX2) ranged from 0.573 to 0.667; the sensitivity of each gene ranged from 29.70 to 51.49% and the specificity ranged from 73.33 to 93.33%, if they were used separately to diagnose stage I NSCLC. The methylation frequencies of the other 6 genes (ALX1, PDGFRA, PITX2, HPP1, OC2 and SMPD3) had no significant difference between tumors and controls, ranging from 24.75 to 59.41% in stage I NSCLC, and from 56.67 to 90.00% in non-cancerous lung diseases (Table III).

### Expression of 8 pathological markers in stage I NSCLC

The positive expression rates of CHGA and SYN were the lowest among the 8 protein (both 3.26%, 3/92), while that of EMA was the highest (100%, 92/92). The positive rates of the other 5 pathological markers were: p21 (8.70%, 8/92), VEGF (28.26%, 26/92), EGFR (29.35%, 27/92), HER-2 (39.13%, 36/92) and p53 (42.39%, 39/92).

### Correlation between the methylation status of the 7 genes and the clinical characteristics of NSCLC

**Figure 1:**
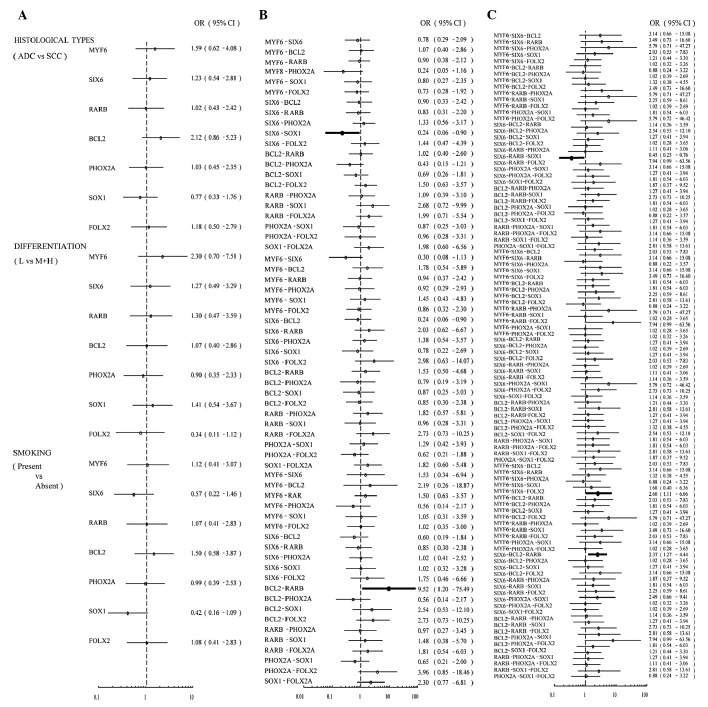
Correlation between the methylation status of 7 genes (singly and combined) and the NSCLC clinical characteristics of NSCLC. Univariate logistic-regression analysis was used. L vs. M+H, low vs. medium+high; OR, odds ratio; 95% CI, 95% confidence interval. NSCLC, non-small cell lung cancer.

The correlations between the methylation status of the 7 genes, individually or combined, with each of the clinical characteristics was primarily assessed by univariate analysis, and the result was displayed in the form of a forest plot (Fig. 1). The methylation status of each of the 7 genes individually had no association with histological types, degree of differentiation or smoking. Next, the correlation between the co-methylation of two genes with the clinical characteristics was assessed in 21 possible pairs of genes. The co-methylation of SIX6 and SOX1 was negatively associated with adenocarcinoma (ADC) with an odds ratio (OR) of 0.24 (95% CI, 0.06–0.90). The co-methylation of BCL2 and RARB was associated with smoking (OR, 9.52; 95% CI, 1.20–75.49). We also analyzed the correlations between co-methylation of three genes with the clinical characteristics. The co-methylation of SIX6, RARB and SOX1 occurred less frequently in adenocarcinoma (ADC) than in squamous cell carcinoma (OR, 0.45; 95% CI, 0.25–0.76). The co-methylation of MYF6, SIX6 and FOLX2 (OR, 2.60; 95% CI, 1.11–6.66) or the co-methylation of SIX6, BCL2 and RARB(OR, 2.37; 95% CI, 1.27–4.44) were associated with smoking (Fig. 1).

To verify these correlations, multivariate regression models were established. These indicated that the co-methylation of SIX6 and SOX1, as well as the co-methylation of SIX6, RARB and SOX1, was negatively associated with ADC; the latter association being more significant (SIX6 and SOX1: OR, 0.243; 95% CI, 0.06–0.98; P=0.045; SIX6, RARB and SOX1: OR, 0.008; 95% CI, 0.001–0.149; P=0.007). The association between the co-methylation of SIX6, BCL2 and RARB and smoking has also been validated (OR, 3.09; 95% CI, 1.20–7.95; P=0.019). However, the association beween smoking and the co-methylation of BCL2 and RARB, or the co-methylation of MYF6, SIX6 and FOLX2, had no statistical significance (P>0.05; Table IV).

### A panel of 4 genes for the diagnosis of stage I NSCLC

Combination of several markers is a common strategy to improve diagnostic sensitivity in studies of clinical biomarkers. In this study, the most outstanding gene for the diagnosis of stage I NSCLC, MYF6, was found to be methylated in 65 of the 101 cases of patients with stage I NSCLC, displaying a sensitivity of 64.36%; and the methylation of MYF6 was also found in 2 of the 30 cases of patients with non-cancerous lung diseases, displaying a specificity of 93.3%. In the 36 cases of stage I NSCLC patients without MYF6 methylation, the methylation frequency of SIX6 was 41.67% (15/36), the highest among the 6 genes other than MYF6. Therefore, we made the first combination of MYF6 and SIX6 for the diagnosis of stage I NSCLC. The sensitivity was improved to 79.21%, while the specificity was dropped to 90.00%. However, the AUC of the ROC curve for the combination of MYF6 and SIX6 was 0.774 (P<0.0001; 95% CI, 0.681–0.866), higher than MYF6 alone, which meant that the combination of MYF6 and SIX6 was superior to MYF6 alone in diagnostic power. The methylation of BCL2 was detected in 8 of the 21 cases without methylation of either MYF6 or SIX6; more frequently than the other 4 genes, thus we made the second combination to form a 3-gene panel (MYF6, SIX6 and BCL2). The sensitivity, specificity and AUC were 87.13%, 86.67% and 0.812 (P<0.0001; 95% CI, 0.717–0.906), respectively. Using this method we analyzed a total of 6 panels of genes. The AUC of the 4-gene panel (MYF6, SIX6, BCL2 and RARB) was the largest among the them, and thus made it the best combination of markers in this study. The sensitivity, specificity and AUC of the 4-gene panel were 93.07%, 86.67% and 0.874 (P<0.0001; 95% CI, 0.787–0.960), respectively (Table V).

### Correlations between the expression of pathological markers and gene methylation in stage I NSCLC

**Figure 2:**
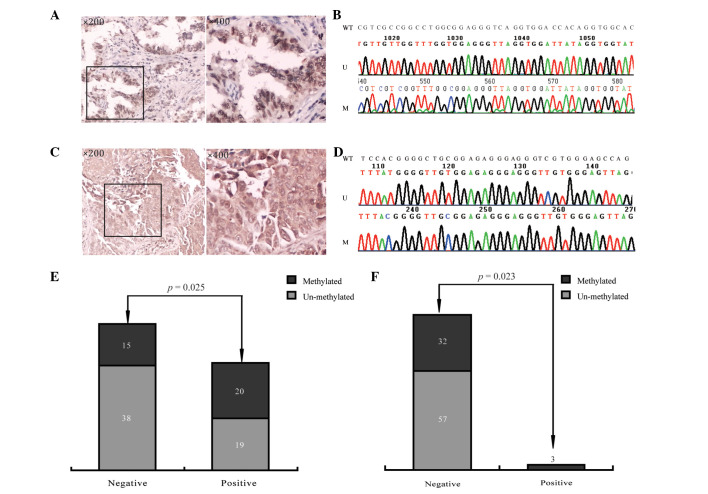
Analysis of the expression data of pathological markers with the DNA methylation status. (A) Staining patterns of P53 in stage I NSCLC by immunohistochemistry. (B) Sequencing verification of both methylated and unmethylated targeted regions of BCL2. (C) Staining patterns of CHGA in stage I NSCLC by immunohistochemistry. (D) Sequencing verification of both methylated and unmethylated targeted regions of PHOX2A. (E) Methylation status of BCL2 in P53-positive and P53-negative stage I NSCLC patients. (F) Methylation status of PHOX2A in CHGA-positive and CHGA-negative stage I NSCLC patients. NSCLC, non-small cell lung cancer; WT, wide-type; U, unmethylated; M, methylated.

We analyzed the data and aimed to explore whether the expression of each protein was associated with the methylation status of any of the 7 genes. We found that the expression of p53 was positively associated with the methylation of BCL2 (P=0.025) and the expression of CHGA was positively associated with the methylation of PHOX2A (P=0.023; Fig. 2 and Table VI).

## Discussion

This study showed that 7 genes (MYF6, SIX6, SOX1, RARB, BCL2, PHOX2A and FOLX2) were frequently methylated in 101 cases of patients with stage I NSCLC, while rarely methylated in 30 patients with non-cancerous lung diseases. A panel of 4 genes (MYF6, SIX6, BCL2 and RARB) was able to diagnose stage I NSCLC from non-cancerous lung diseases with a sensitivity of 93.07% and a specificity of 86.67%.

RARB and BCL2 have already been found to be hypermethylated in NSCLC (4,5). The protein encoded by SOX1 acts as transcription factor and plays a part in the regulation of embryonic development and in the determination of cell fate. Although the methylation of SOX1 have been found to be associated with genitourinary tumors, including cervical cancer, prostate cancer and ovarian cancer, this is the first time that methylation of SOX1 has been found in NSCLC (6–8). It has been reported that SOX1 antibodies are common in the serum of patients with small cell lung carcinoma (SCLC) and may be serve as specific serological markers (9). The methylation of 4 genes, MYF6, SIX6, PHOX2A, FOLX2, has never previously been reported in any types of cancer. MYF6 (12q21) is involved in muscle differentiation, SIX6 (14q23.1) is thought to be involved in eye development, and PHOX2A (11q13.2) is vital for development of the autonomic nervous system (10), FOXL2, as a forkhead transcription factor, may be involved in ovarian development and function. Further studies in the functions of these gene may help to reveal the mechanism of malignant transformation of non-small-cell lung cells.

We found that the co-methylation of SIX6 and SOX1 correlated with squamous cell carcinoma (SCC), while the methylation of neither of them individually demonstrated an association with SCC, which may imply that the methylation of these two genes had a superimposed effect on the development of SCC. The co-methylation of BCL2, RARB and SIX6, but not the methylation of either single gene, was associated with smoking. We postulated that cigarette smoking may cause the methylation of the 3 genes through a common pathway.

To explore the possible pathway through which gene methylation promotes the carcinogenesis and progression of NSCLC, we investigated the expression of 8 classical components (VEGF, HER-2, P53, P21, EGFR, CHGA, SYN and EMA) of common oncogenic pathways in 92 cases of stage I NSCLC using immuno histochemistry. Analyzing the expression data with the DNA methylation status in this study, we found that the expression of P53 and CHGA were positively associated with the methylation of BCL2 and PHOX2A, respectively. Bcl2, encoded by the gene BCL2, as a critical pro-survival member of Bcl2 protein family which regulates apoptosis, is usually considered to be a downstream target of p53 and can be negatively regulated by p53 (11,12). The overexpression of Bcl2 has been found in numerous types of cancer, including breast cancer, prostate cancer, B-cell lymphoma and colorectal adenocarcinoma (13). However, recently, the hypermethylation of BCL2 gene has been reported in certain types of cancer, such as prostate cancer (14). Furthermore, the methylation of BCL2 was found to be associated with tumor invasion in peripheral pulmonary adenocarcinoma (5). In this study, the methylation frequency of BCL2 was significantly higher in lung tissues of stage I NSCLC, than in non-cancerous lung diseases, and its methylation was positively associated with the expression of P53 in stage I NSCLC. Wang *et al* reported that wild-type p53 negatively regulated DNMT1 expression through interaction with specificity protein 1 (Sp1) protein (6). Since DNA methylation was usually accompanied by gene silencing, the role of BCL2 in the development and progression of cancer was complicated and further studies are warranted. The methylation of PHOX2A demonstrated positive association with the expression of CHGA. CHGA protein was thought to be a tumor marker in neuroendocrine tumors (NETs), but high expression of CHGA was also found in several other types of solid tumor, such as small cell lung cancer (15), and higher expression of CHGA was associated with higher pathological stage in prostate cancer (16). The function of PHOX2A in carcinogenesis of NSCLC may be correlated with CHGA.

Tumorigenesis is an intricate process, involving a variety of genetic and epigenetic aberrations. Even a single tumor-related gene may simultaneously display several types of abnormalities, and contribute to tumorigenesis through several different ways. In this study, 5 genes were for the first time found to be hypermethylated in NSCLC, and the function of those genes and how they act in the carcinogenesis of NSCLC is worth further exploration.

## Figures and Tables

**Table I:** Clinical profile of the stage I NSCLC cancer patients and non-cancerous lung diseases controls.

Characteristics	Stage I NSCLC (n=101)	Non-cancerous lung lesions (n=30)
Gender		
Female	37	9
Male	64	21
Age (years)		
31–40	1	0
41–50	9	7
51–60	32	12
61–70	39	6
71–80	20	5
Range	32–79	42–76
Median	61.28±8.92	60.48±7.90
Histological types		
Squamous cell carcinoma	24	
Adenocarcinoma	48	
Adenosquamous carcinoma	14	
Others	15	
Types of non-cancerous lung lesions		
Pulmonary tuberculosis		6
Bronchiectasis		5
Pulmonary abscess		6
Organizing pneumonia		2
Pulmonary sclerosing hemangioma		3
Pulmonary giant lymph node hyperplasia		2
Pulmonary hamartoma		3
Pulmonary sequestration		1
Pulmonary inflammatory pseudotumor		2

NSCLC, non-small cell lung cancer.

**Table II:** Primer list for methylation-specific PCR.

Gene name	GenBank No.	Sense 5′-3′	Antisense 5′-3′	Size (bp)
ALX1	NC_000012.11	TTTTTTGGAGTACGTTATGGAGAC	AACGCACGTAATACTCGACG	116
BCL2	NG_009361.1	GAAGTCGTCGTCGGTTTG	CCCGCACCGAACATC	183
FOXL2	NG_012454.1	GTTATAATATTTTTTCGGTTGTTCG	CTAACTCCACGACCTATACTCGAT	211
HPP1	AF242221	AAGAGGGGCGTTAGTTCG	CGCTCGCAAACGCTAA	158
MYF6	NG_021392.1	GGAAATGCGTATTCGGTTC	CGAACCCCCTAAAATAATCG	182
OC2	NC_000018.9	CGGGTTCGTAGGTGGTTAC	TCCACGATTTTAAATTCCGA	177
PDGFRA	NG_009250.1	CGTCGCGTTGTTTTATTTTC	AATCGACCTTACGCCTATCG	160
PHOX2A	NG_008169.1	AGGGATAGTTATAAGCGCGG	AAAAATACAAAATCGTATAAACCTCG	211
PITX2	NG_007120.1	GATCGTTAGTCGCGTAGTCG	TCCAACTTTCTCGCTCGAT	177
RARB	NM_016152	TCGAGAACGCGAGCGATTCG	GACCAATCCAACCGAAACGA	146
SIX6	NG_008203.1	TTAGTAGTTAGGCGTTGGGATC	CCTCTCGAAATAATTACTTTACCG	150
SMPD3	NC_000016.9	TCGTAGGATTTTCGAAGGATC	CATCACCGACGAATATAATCG	160
SOX1	NC_000013.10	GGTATTGGCGAATTTTAGTGTAC	AAAAAAACGCTCCCTTAAACG	135

**Table III:** Diagnosis performance of the 13 methylation targets in stage I NSCLC versus non-cancerous lung diseases.

	Stage I NSCLC		Non-cancerous lung diseases						
Target	Sensitivity (%)	pos./total	Specificity (%)	pos./total	AUC	(95% CI)	PPV	NPV	P-value
MYF6	64.36	65/101	93.33	2/30	0.704	0.613–0.795	0.64	0.93	<0.0001
SIX6	51.49	52/101	90.00	3/30	0.650	0.557–0.743	0.51	0.90	0.0007
SOX1	50.50	51/101	73.33	8/30	0.573	0.475–0.671	0.51	0.70	0.0213
RARB	47.52	48/101	96.67	1/30	0.667	0.576–0.757	0.48	0.97	<0.0001
BCL2	37.62	38/101	90.00	3/30	0.613	0.515–0.712	0.38	0.90	0.0035
PHOX2A	35.64	36/101	93.33	2/30	0.624	0.526–0.722	0.36	0.93	0.0013
FOLX2	29.70	30/101	93.33	2/30	0.610	0.507–0.714	0.30	0.93	0.0082
ALX1	59.41	60/101	56.67	13/30	N	N	0.59	0.57	0.1447
PDGFRA	37.62	38/101	70.00	9/30	N	N	0.38	0.70	0.5195
PITX2	34.65	35/101	83.33	5/30	N	N	0.35	0.83	0.0724
HPP1	32.67	33/101	56.67	13/30	N	N	0.33	0.57	0.2864
OC2	24.75	25/101	86.67	4/30	N	N	0.25	0.87	0.2197
SMPD3	24.75	25/101	90.00	3/30	N	N	0.26	0.90	0.0819

NSCLC, non-small cell lung cancer; AUC, area under curve; PPV, positive predictive value; NPV, negative predictive value.

**Table IV:** Multivariate analysis of the correlation between gene methylation and clinical characteristics of NSCLC.

Case	Genes	Status	No. of methylations	No. of cases	OR	95% CI	P-value
ADC/SCC	SIX6-SOX1	Negative	53	2			
		Positive	19	17	0.24	0.06–0.98	0.045
	SIX6-RARB-SOX1	Negative	60	0			
		Positive	12	12	0.01	0.001–0.149	0.007
Smoking	BCL2-RARB	Negative	85	1			
		Positive	16	15	9.16	0.99–84.45	0.051
	SIX6-BCL2-RARB	Negative	93	1			
		Positive	8	7	3.09	1.20–7.95	0.019
	MYF6-SIX6-FOLX2	Negative	87	1			
		Positive	14	13	8.11	0.83–78.50	0.071

NSCLC, non-small cell lung cancer; ADC, adenosquamous carcinoma; SCC, squamous cell carcinoma; OR, odds ratio; CI, confience interval.

**Table V:** Diagnostic performance of different panels of genes in stage I NSCLC, using patients with non-cancerous lung diseases as controls.

	NSCLC (n=101)		Non-cancerous lung diseases (n=30)						
	Sensitivity (%)	pos./total	Specificity (%)	pos./total	AUC	95% CI	PPV	NPV	P-value
MYF6	64.36	65/101	93.33	2/30	0.681	0.587–0.774	0.970	0.438	<0.0001
MYF6,SIX6	79.21	80/101	90.00	3/30	0.774	0.681–0.866	0.964	0.571	<0.0001
MYF6,SIX6,BCL2	87.13	88/101	86.67	4/30	0.812	0.717–0.906	0.957	0.667	<0.0001
MYF6,SIX6,BCL2,RARB	93.07	94/101	86.67	4/30	0.874	0.787–0.960	0.961	0.798	<0.0001
MYF6,SIX6,BCL2,RARB, PHOX2A	94.06	95/101	83.33	5/30	0.868	0.792–0.964	0.950	0.807	<0.0001
MYF6,SIX6,BCL2,RARB, PHOX2A,SOX1	96.04	97/101	63.33	11/30	0.836	0.731–0.940	0.898	0.826	<0.0003

NSCLC, non-small cell lung cancer; AUC, area under curve; PPV, positive predictive value; NPV, negative predictive value.

**Table VI:** Correlations between the expression of pathological markers with gene methylation in stage I NSCLC.

	CHGA	EGFR	EMA	HER-2	P21	P53	SYN	VEGF
MYF6	N	N	N	N	N	N	N	N
SIX6	N	N	N	N	N	N	N	N
RARB	N	N	N	N	N	N	N	N
BCL2	N	N	N	N	N	0.025	N	N
PHOX2A	0.023	N	N	N	N	N	N	N
SOX1	N	N	N	N	N	N	N	N
FOLX2	N	N	N	N	N	N	N	N

NSCLC, non-small cell lung cancer; N, no sense.
